# Higher National Education

**Published:** 1903-09-19

**Authors:** 


### The Hospital.

A Journal of

The Medical Sciences and Hospital Administration.

Vol. XXXIV.—No. 886. SEPTEMBER 19, 1903.

### HIGHER NATIONAL EDUCATION.

The weighty indictment of our system, or want of system, of higher national education, which formed the subject of Sir Norman Lockyer's presidential address to the British Association, unquestionably calls for the careful consideration of all the more thoughtful classes of the community, and especially, we think, for the consideration of the medical profession, and for the exertion of all the influence which its members can bring to bear upon public opinion for the correction or removal of the evils which Sir Norman pointed out. Briefly stated, his position is that we are far outstripped by other nations in our provision for what may be called higher education, and especially in our provision for bringing it within the reach of the children of families of slender means, and that we are consequently fast being outstripped in our power of bringing the results of such education to bear upon the improvement of our industries. Something like this has often been said before, but, as far as we remember, never by a speaker so entirely conversant with the subject; nor has the statement ever before been so copiously illustrated by facts displaying the practice alike of our rivals and of ourselves. Sir Norman tells us that we have in the United Kingdom 13 universities, as against 134 in the United States of America, and 22 in Germany. Twelve English universities have received during 60 years, as gifts from private persons, less than four millions sterling; while the universities and colleges of the United States received nearly seven millions in the years 1898–1900, and more than forty millions within a few years preceding. Our State endowments for the whole of our universities amount to about £155,000 a year, or less by about £14,000 than is given by Germany to the University of Berlin alone. One net result of these differences is that our teachers are rendered largely dependent upon the fees of students for their remuneration; and this dependence entails a necessity for the maintenance of a scale of fees which closes the doors of the class rooms against the comparatively poor. We should expect Sir Noiman to maintain, moreover, that the education actually given is not of a character to prepare the comparatively poor for the positions which they ought to hold in the ranks of industrial competition. It is neither sufficiently scientific nor sufficiently practical, and its tendency is to turn out either elegant scholars who are ignorant of the meaning of exactness, or profound mathematicians who do not stoop to the problems of the engineering shop, or a rank and file of graduates who know a little of several things but know nothing well, and who are not exempt from the weakness of fancying themselves superior to anything so dirty and so commonplace as mechanical industry. In the meanwhile, our exports are being surpassed alike in quality, in price, and in adaptiveness to the purposes for which they are required.

In no department is this want of education more conspicuous than in the applications of science to the prevention or cure of disease. At the time of the first Jubilee of Queen Victoria it was declared, and on good grounds, that the advances during 50 years in medicine and surgery due to British practitioners were more important and more considerable than those due to the practitioners of all other nations put together. We greatly doubt whether this position is likely to be maintained for the future, in the face of a declaration by the Royal Colleges of Physicians and Surgeons of London and of England that the "scientific" education of an inferior boys' school is a sufficient preparation for the study of medicine. The question raised by Sir Norman Lockyer may not have much practical bearing upon the status of existing British practitioners. It may, nevertheless, have a very important bearing alike upon the status, the efficiency, and the remuneration of their successors; and it will be an evil day for the profession if good ground ever arises for thinking its members in this country inferior in skill and knowledge to the foreigners who, in that case, will speedily appear upon the scene as competitors. We do not think the danger entirely visionary, and, in whatever degree it may be real, it affords to the individual members of the profession a real basis of interest. We would urge upon them the prudence of assisting to bring the views of Sir Norman Lockyer home to the perceptions of their patients, and especially to those around whom boys of some promise are growing up. Every such boy may easily be made the text of a convincing sermon, because its doctrines will appeal to parental ambition and solicitude. If such sermons are preached in many homes, they will hardly fail to do much towards the creation of a public opinion which will in its turn react alike upon Chancellors of the Exchequer and upon wealthy private individuals. It is probably true that the man of great genius will overcome all difficulties, and that facilities will not greatly help, nor impediments greatly hinder, the natural course of his career. However this may be, it is indubitably true that the man of only moderate parts may be almost indefinitely helped by education; or, in other words, that he may be fitted to fill with credit and usefulness a position in which he would otherwise have been a failure.

